# The farnesyl transferase inhibitor (FTI) lonafarnib improves nuclear morphology in ZMPSTE24-deficient fibroblasts from patients with the progeroid disorder MAD-B

**DOI:** 10.1080/19491034.2023.2288476

**Published:** 2023-12-05

**Authors:** Kamsi O. Odinammadu, Khurts Shilagardi, Kelsey Tuminelli, Daniel P. Judge, Leslie B. Gordon, Susan Michaelis

**Affiliations:** a Department of Cell Biology, The Johns Hopkins School of Medicine, Baltimore, MD, USA; b The Progeria Research Foundation, Peabody, MA, USA; c Department of Medicine, Medical University of South Carolina, Charleston, SC, USA; d Department of Anesthesiology, Critical Care and Pain Medicine, Boston Children’s Hospital and Harvard Medical School, Boston, MA, USA; e Department of Pediatrics, Division of Genetics, Hasbro Children’s Hospital and Warren Alpert Medical School of Brown University, Providence, RI, USA

**Keywords:** Atypical progeroid syndrome, *LMNA-C588R*, *LMNA-D325N*, *LMNA-E138K*, *LMNA-M540T*, *LMNA-R644C*, mandibuloacral dysplasia-type B, prelamin A, *ZMPSTE24-L425P*, *ZMPSTE24-P248L*

## Abstract

Several related progeroid disorders are caused by defective post-translational processing of prelamin A, the precursor of the nuclear scaffold protein lamin A, encoded by *LMNA*. Prelamin A undergoes farnesylation and additional modifications at its C-terminus. Subsequently, the farnesylated C-terminal segment is cleaved off by the zinc metalloprotease ZMPSTE24. The premature aging disorder Hutchinson Gilford progeria syndrome (HGPS) and a related progeroid disease, mandibuloacral dysplasia (MAD-B), are caused by mutations in *LMNA* and *ZMPSTE24*, respectively, that result in failure to process the lamin A precursor and accumulate permanently farnesylated forms of prelamin A. The farnesyl transferase inhibitor (FTI) lonafarnib is known to correct the aberrant nuclear morphology of HGPS patient cells and improves lifespan in children with HGPS. Importantly, and in contrast to a previous report, we show here that FTI treatment also improves the aberrant nuclear phenotypes in MAD-B patient cells with mutations in *ZMPSTE24* (P248L or L425P). As expected, lonafarnib does not correct nuclear defects for cells with lamin A processing-proficient mutations. We also examine prelamin A processing in fibroblasts from two individuals with a prevalent laminopathy mutation *LMNA*-R644C. Despite the proximity of residue R644 to the prelamin A cleavage site, neither R644C patient cell line shows a prelamin A processing defect, and both have normal nuclear morphology. This work clarifies the prelamin A processing status and role of FTIs in a variety of laminopathy patient cells and supports the FDA-approved indication for the FTI Zokinvy for patients with processing-deficient progeroid laminopathies, but not for patients with processing-proficient laminopathies.

## Introduction

**Figure 1.**
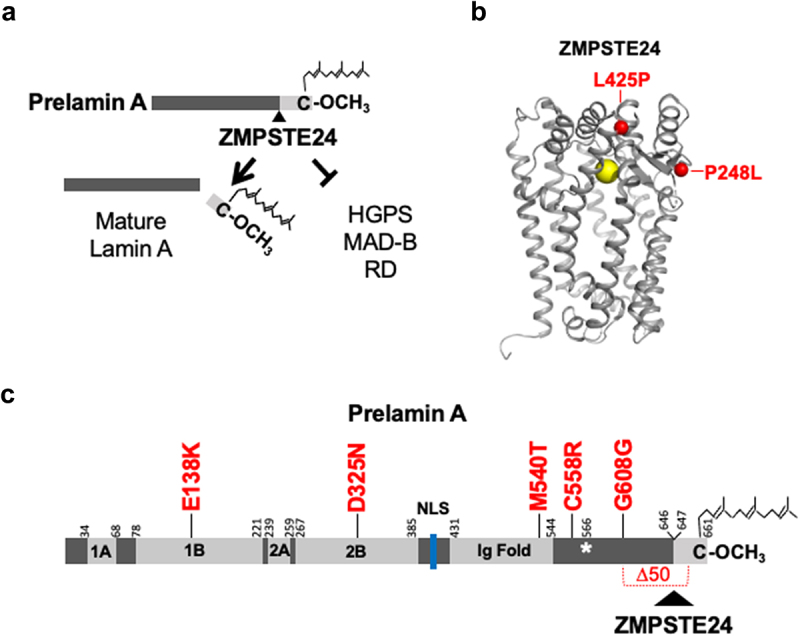
Prelamin A processing and sites of *ZMPSTE24* and *LMNA* mutations in patient cells used in this study. (a) Farnesylated and carboxyl methylated prelamin A undergoes proteolytic cleavage of the C-terminal 15 amino acids (light gray box) by ZMPSTE24 to produce non-farnesylated mature lamin A (left). When processing is disrupted, permanently farnesylated disease-specific forms of prelamin A accumulate and cause the progeroid laminopathy diseases HGPS, MAD-B, and RD. The farnesyl (jagged line) and carboxyl methyl group (OCH_3_) on the C-terminal cysteine, C661, are indicated; the ZMPSTE24 prelamin A cleavage site residues 646 and 647 is indicated by a black triangle. (b) The ZMPSTE24 ribbon structure [13] was generated in Pymol. Zinc (yellow) is at the catalytic center. The position of amino acid substitutions of the MAD-B patient mutations in this study are marked in red. (c) A schematic of prelamin A is shown. The *LMNA* mutations examined in Figures 1–3 of this study are indicated in red. The HGPS mutation G608G used as a control in this study is indicated; it results in a 50 amino acid in-frame internal deletion (∆50) in brackets and includes the ZMPSTE24 processing site. The central rod domain of prelamin A is comprised of four α-helical segments (1A, 1B, 2A, and 2B), the nuclear localization signal (NLS, blue), the immunoglobulin fold (ig Fold) domain, and the C-terminal farnesylated and carboxyl methylated cysteine are indicated. The ZMPSTE24 cleavage site between residues Y646 and L647, which are numbered, is shown with a triangle. Other numbers indicate domain start and endpoints, as shown by Simon and Wilson [14]. Note that prelamin A is not shown to scale. The asterisk denotes the final residue shared by lamin A and lamin C (residue 566); lamin C is a *LMNA* splice variant and has six additional unique amino acids not indicated here.

Among the progeroid laminopathies are a group of related diseases caused by defective post-translational processing of prelamin A, the precursor for the nuclear scaffold protein lamin A [1–6]. The *LMNA* gene encodes lamin A and its splice variant lamin C, which together with lamins B1 and B2, are the major components of the nuclear lamina meshwork underlying the inner nuclear membrane. The lamins multimerize to form homopolymeric intermediate filaments that provide structural support for the nucleus and influence numerous nuclear functions [7–11]. Lamin A is synthesized as a precursor, prelamin A, that contains a C-terminal CAAX motif that undergoes a series of post-translational modifications to yield farnesylated and carboxyl methylated prelamin A. Nearly all CAAX proteins retain these modifications, which are required for their membrane localization and proper functioning [12]. In contrast, however, prelamin A is unique among CAAX proteins, as it undergoes a final proteolytic processing step mediated by the zinc metalloprotease ZMPSTE24 that removes the modified C-terminal 15 residues of lamin A (Figure 1(a)) [1,5,15,16]. ZMPSTE24 cleavage is rapid and efficient so that essentially no prelamin A is generally detected *in vivo* and the cleaved portion appears to be rapidly degraded. The reason why cells modify prelamin A only to rapidly cleave off the modified C-terminus is not understood, but this proteolytic cleavage is clearly critical for normal health and longevity. The failure to do so, either due to mutations in *LMNA* that alter or delete residues adjacent to the prelamin A cleavage site, or mutations in *ZMPSTE24* itself that diminish its function, results in permanently farnesylated forms of lamin A that drive the progeroid disorders discussed below.

The rarest but best studied of the processing-deficient progeroid laminopathies is Hutchinson Gilford progeria syndrome (HGPS). HGPS is characterized by premature aging starting at about 1 year of age, with failure to thrive, bone dysplasia, generalized lipodystrophy, alopecia, and progressive atherosclerosis that results in death of HGPS patients from heart attack or stroke at a median age of ~15 years when untreated [17–20]. A characteristic feature of fibroblasts from HGPS patients is aberrant nuclear morphology [21]. Nearly all HGPS patients harbor a sporadic autosomal dominant *LMNA* mutation that, through altered splicing, generates an internally deleted (∆50) version of prelamin A called progerin [22,23]. Progerin retains the CAAX motif of prelamin A but lacks the ZMPSTE24 cleavage site. The resulting permanently farnesylated and carboxyl methylated ∆50 form of lamin A acts in a dominant negative fashion to drive disease phenotypes [24]. Restrictive dermopathy (RD) and mandibuloacral dysplasia-type B (MAD-B) are recessive progeroid laminopathies that result from mutations in *ZMPSTE24* and lead to accumulation of permanently farnesylated and carboxyl methylated full-length prelamin A [1,25,26]. RD is due to complete loss-of-function mutations in both copies of *ZMPSTE24* and is lethal before or just after birth, due to severe developmental defects [27,28]. MAD-B is caused by a partial loss-of-function allele in one copy of *ZMPSTE24* and a null allele in the other, or two partial loss of function alleles [25,29–31]. MAD-B is generally less severe than HGPS and the lifespan of reported patients varies from the teens to the 40s. *ZMPSTE24* point mutations can result in varying levels of residual proteolytic activity which scale inversely with disease severity, and can also cause protein instability [26,32,33]. MAD-B is characterized by growth retardation, partial lipodystrophy, and bone abnormalities, including hypoplasia of the mandible and clavicles, as well as osteolysis of the distal phalanges, and many patients show dental crowding [25,29,34]. Taken together, RD, MAD-B, and HGPS form a clinical continuum of related phenotypes.

Studies to inhibit farnesylation using farnesyl transferase inhibitors (FTIs) were performed within a few years after HGPS was mapped to *LMNA*. In those studies, FTIs were found to block aberrant nuclear morphology and improve phenotypes in tissue culture cells expressing progerin, in HGPS patient cells, and in HGPS mouse models [35–42]. Subsequently, clinical studies have shown that the FTI lonafarnib improves the lifespan of children with HGPS by ~2.5 years [43–45] and is accompanied by a 35–62% decrease in plasma progerin [46]. By extension, it seems likely that lonafarnib might also benefit MAD-B patients, since their disease also results from a permanently farnesylated form of lamin A, *i.e*., full-length prelamin A (rather than the ∆50 progerin version of prelamin A found in HGPS). However, MAD-B patient cells and cellular models of MAD-B have been less well studied than those of HGPS patients, and the existing literature presents some perplexing contradictions. On the one hand, tissue culture cells expressing a full length uncleavable form of GFP-prelamin A show improved nuclear morphology with FTI treatment [38] as has also been shown for fibroblasts from an RD patient [39]. Furthermore, the disease phenotypes of *Zmpste24*-/- mice dramatically improve with administration of FTIs [47]. However, a study by Akinci *et al.* [48] with MAD-B patient fibroblasts carrying the *ZMPSTE24*-P248L mutation concluded there was no improvement of nuclear morphology with FTI treatment.

In this report, we examine a variety of patient fibroblasts, including those from 3 patients with MAD-B due to mutations in *ZMPSTE24* (P248L and L425P) and those from patients with atypical progeroid syndromes (APS) whose mutations map in *LMNA* (D325N, M540T, C558R, and E138K), but at a distance from the processing site. Clinical Information for patients not published previously is provided. We confirm that, as expected, the APS mutations do not affect prelamin A processing. All of these patient cells exhibit aberrant nuclear morphology, but importantly, we find that only those from MAD-B, and not those from APS patients, show improved nuclear morphology with FTI treatment, and thus are likely to benefit from FTI therapy. Our findings contrast with those of the previous study which reported that nuclear morphology of MAD-B patient with the *ZMPSTE24*-P248L mutation does not improve upon FTI treatment [48]. We also provide here, for the first time, an analysis of prelamin A processing in fibroblasts from two unrelated patients with one of the most prevalent laminopathy mutations, *LMNA*-R644C, which alters an arginine residue near, but not at, the ZMPSTE24 processing site in prelamin A between residues Y646 and L647. Our results indicate that processing is proficient and nuclear morphology is normal in the R644C patient cells, suggesting that FTIs would not benefit patients with this mutation.

## Materials and methods

### Patient fibroblasts and growth conditions

Primary fibroblasts from laminopathy patients and an unaffected individual used in this study are listed in Table 1 and a brief description of the clinical features of patients not previously published is provided in the Supplemental Material. Cells were obtained as frozen stocks from the indicated sources, at passages 7–9 for cells from The Progeria Research Foundation (PRF) Cell and Tissue Bank (http://www.progeriaresearch.org), passage 2 for R644C-1 cells, and passage 6 for L647R cells. Cells were grown in DMEM with high glucose without l-glutamine, supplemented with 15% fetal bovine serum and 1X penicillin–streptomycin–glutamine.

**Table 1.** Primary patient fibroblasts used in this study.

Patient Fibroblast Mutation	Patient Cell Line Name	Genotype^a^	Clinical Diagnosis	Source^b^	Reference^c^
**Wild-type (WT)**	HGFDFN168	WT	Unaffected	PRF	[49]
***LMNA*****Mutations**					
E138K	PSADFN485	*LMNA* c.412 G>A	APS	PRF	This study
D325N	PSADFN363	*LMNA* c.973 G>A	APS	PRF	This study
M540T^d^	PSADFN257	*LMNA* c.1619T>C homozygous	APS/ (MAD-A)	PRF	[50]
C588R	PSADFN412	*LMNA* c.1762T>C	APS	PRF	[51]
G608G	HGADFN167	*LMNA* c.1824C>T	HGPS	PRF	[49]
R644C-1^a^	PSADFN542	*LMNA* c.1930C>T	Laminopathy/ Cardiomyopathy	PRF	This study
R644C-2^b^	R644C-2	*LMNA* c.1930C>T	Laminopathy/ Cardiomyopathy	D.P. Judge	This study
L647R	L647R	*LMNA* c.1933T>G	MAD-B-like	H. Worman	[52]
***ZMPSTE24*****Mutations**					
P248L–1^e^	PSADFN317	*ZMPSTE24* c.743C>T, (p.Pro248Leu) c.1349 G>A (p.Trp450Stop)	MAD-B	PRF	[34]
P248L–2^e^	PSADFN318	*ZMPSTE24* c.743C>T, (p.Pro248Leu) c.1349 G>A (p.Trp450Stop)	MAD-B	PRF	[34]
L425P^f^	PSADFN373	*ZMPSTE24* c.1274C>T, (p.Leu425Pro) homozygous	MAD-B	PRF	This study

^a^Unless otherwise noted, these *LMNA* mutations are dominant; each patient also carries a WT allele (not indicated).

^b^PRF is The Progeria Research Foundation Cell and Tissue Bank (www.progeriaresearch.org).

^c^Clinical information for patients can be found in the indicated reference. For those listed as ‘This Study’ clinical information is provided by The Progeria Research Foundation’s Medical and Research Database or International Patient Registry, and a brief summary is presented in the Supplementary Material. For R644C-2, clinical information in the Supplementary Material was provided by D. P. Judge, as indicated.

^d^This patient has the same M540T mutation on both copies of LMNA due to uniparental disomy.

^e^These *ZMPSTE24* -P248L patients are siblings. They are called 4700.3 and 4700.4 in the reference shown and in Ahmad *et al*. and Akinci *et al*. [34,48].

^f^This patient has the same mutation on both copies of *ZMPSTE24*.

### Preparation of lysates, SDS-PAGE, and western blot analysis

**Figure 2.**
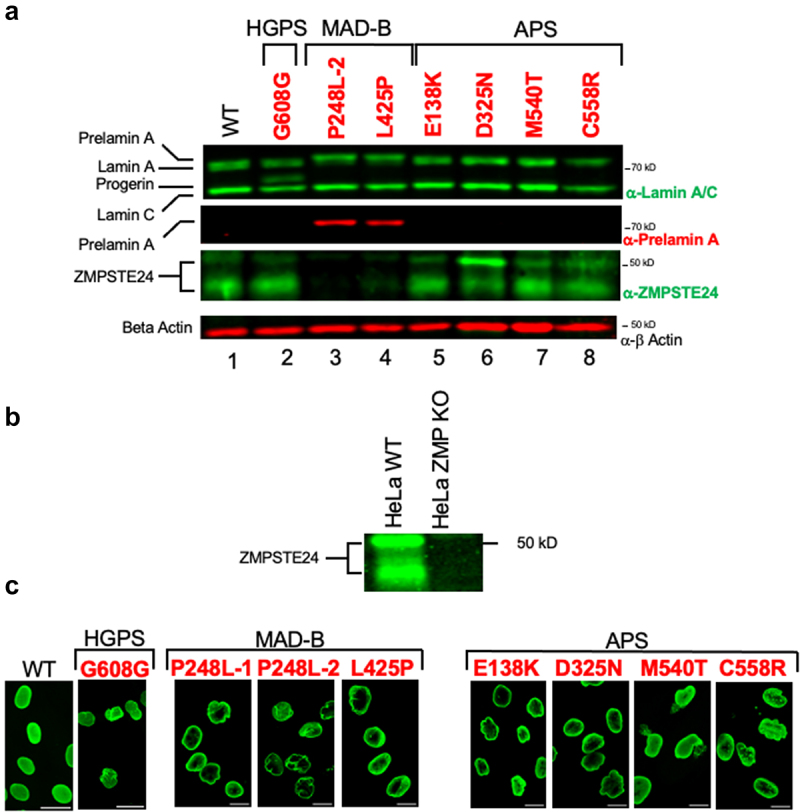
Analysis of prelamin A processing status and nuclear morphology in patient cells used in this study. (a) Immunoblots of protein extracts from WT and HGPS, MAD-B, and APS patient fibroblasts. Extracts from fibroblasts, prepared at passage 12 were analyzed by 10% SDS-PAGE transferred to nitrocellulose, and probed with the indicated antibodies. The panels from top to bottom show lamin A/C, prelamin A, ZMPSTE24, and β-actin staining, as indicated. As discussed in the text, ZMPSTE24 standardly migrates anomalously in SDS-PAGE at ~ 40–50 kD (unlike the calculated MW of 55 kD) as a doublet or smear. (b) Validation of α-ZMPSTE24 antibody (Invitrogen, #PA1 –16,965 lot # UD2763487) used in this study, in extracts from HeLa cells that do (WT) and do not (ZMPSTE24 KO) express ZMPSTE24. (c) Representative immunofluorescence micrographs of WT and patient cells, stained with anti-lamin A/C antibody. Scale bar is 20 µm.

Cell lines were seeded at a density of ~7 × 10^4^ cells/well into a single well of a 6-well plate and allowed to grow for 24 hr. Lysates were prepared in two different ways: For Figure 2, cells were trypsinized (Trypsin-EDTA, Gibco 25200056) for ~5 min and trypsin was quenched with Defined Trypsin Inhibitor (DTI; Gibco, R007100). Cells were then pelleted, and lysed using NP40-Triton lysis buffer (0.6 mM Triton X-100, 5 M NaCl, 1 M Tris pH7.4, 0.5 M EDTA, 0.5 M EGTA, 10% NP40) with 1 μM PMSF and protease inhibitor cocktail (cOmplete Mini Protease Inhibitor tablets, EDTA-free; Sigma, #11836170001), using 1 tablet/10 μl of lysis buffer and rotated for 30 min. Lysates were pelleted, and supernatant was added to an equal volume of 4× Laemmli Sample Buffer (Bio-Rad) with β-mercaptoethanol (BME), heated at 65°C for 10 min, sonicated (3 bursts of 10 sec each, keeping cold in between bursts) and analyzed by SDS-PAGE or stored frozen for the future use. For Figure 4, cells were lysed ‘in well’ using SDS-loading buffer (SDS-LB) (4% SDS, 0.2% Bromophenol blue, 20% glycerol, 0.014% BME, and lysates were heated, sonicated, and stored as above.

**Figure 3.**
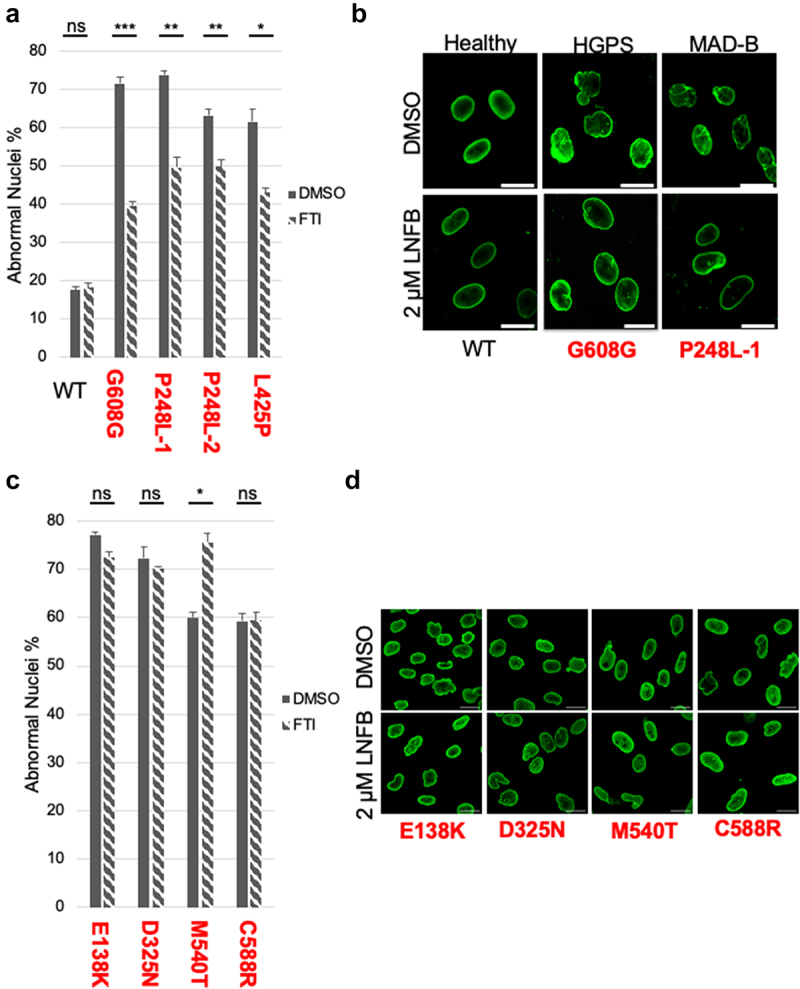
FTI treatment improves nuclear morphology in MAD-B patient cells which have a prelamin A processing defect, but not in APS patient cells in which processing is unaffected. (a, c) Percentage of abnormal nuclear morphology in untreated (vehicle only) or FTI-treated patient cells. Cells were treated with vehicle only or 2 µM lonafarnib for 48 hr. Results from healthy and processing-defective (MAD-B and HGPS) patient cells are shown in (a), and from processing-proficient APS patient cells in (c). For each patient cell line, cell populations were propagated independently (*n* = 3) and ~ 350 nuclei/population for a) or ~ 250 nuclei/population for (c) were counted. Scoring criteria for normal and aberrant nuclear morphology are discussed in the materials and methods. Values plotted represent the mean; error bars indicate the standard error of the mean (SEM). Statistical significance was determined by unpaired, two-tailed *t*-test, with *P*-values indicated as follows: ns, *P* > 0.05; * *P* < 0.05; ** *P* < 0.01; *** *P* < 0.001; ****P < .0001. (b, d) Representative immunofluorescence micrographs of the patient cell lines, untreated or lonafarnib (LNFB)-treated and stained with anti-lamin A/C antibody, are shown. The scale bar is 20 µm.

**Figure 4.**
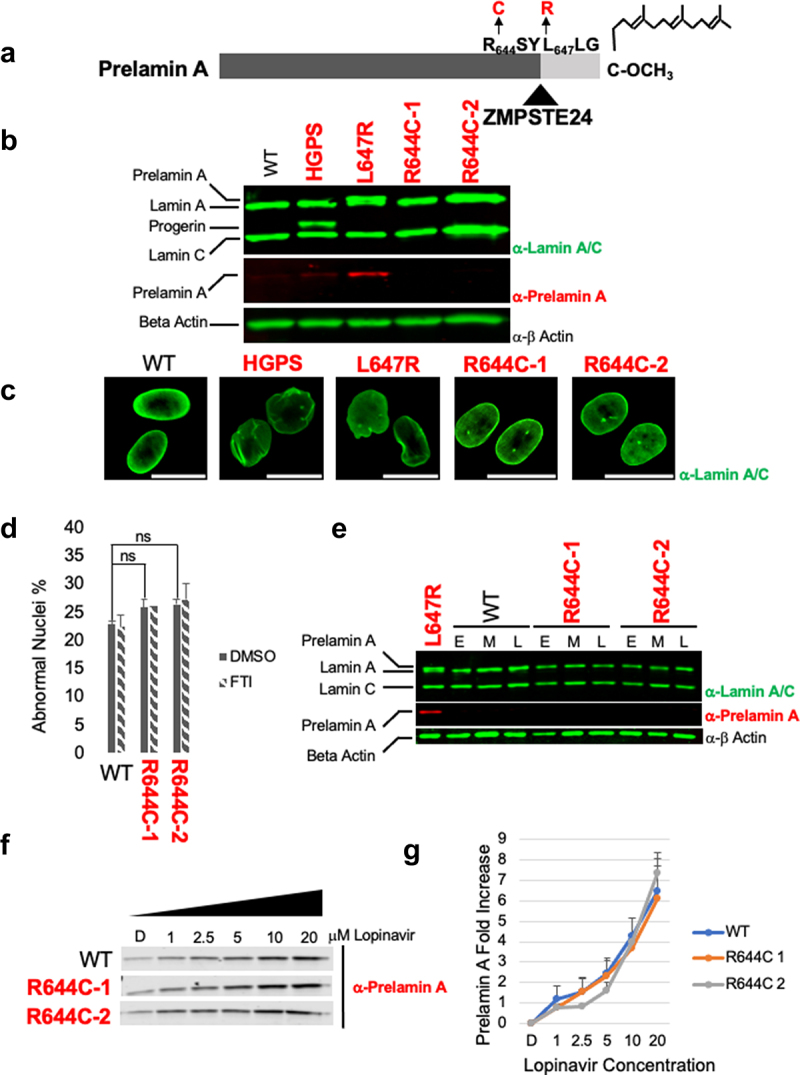
R644C patient cells have no apparent prelamin a processing defect and normal nuclear morphology. (a) The six residues (_644_RSYLLG_649_) in prelamin A that flank the ZMPSTE24 processing site are indicated. The L647R mutation that blocks processing and causes a MAD-B-like disease [52] and the R644C patient mutations (this study) are indicated in red. Other features shown are as in Figure 1. (b) Protein extracts from control (WT) and patient cell lines were analyzed by SDS-PAGE and immunoblotting with anti-lamin A/C, anti-prelamin A and anti-β-actin antibodies, as indicated. (c) Representative immunofluorescence images of control and patient cell lines cell nuclei, stained with anti-lamin A/C antibody. Scale bar is 20 µm. (d) Percentage of abnormal nuclear morphology in untreated (vehicle only) or FTI-treated patient cells are shown, as described in the legend of Figure 3. The difference between abnormal nuclear morphology of WT and R644C-1 and -2 cells is not significant (*P* > 0.05); nor is there a significant difference in abnormal nuclear morphology between untreated and FTI-treated cells for each patient cell line (*P* > 0.05), as determined by unpaired two-tailed *t*-test. (e) Immunoblot of early (E), mid (M), and late (L) passages of control (WT) and R644C patient cells, staining with α-laminA/C and α-prelamin A antibodies. Passages E, M, and L are passage numbers 15, 20, and 26 for WT cells, 15, 23 and 27 for R644C-1 cells, and 15, 20 and 26 for R644C-2 cells. (f) Immunoblot blot of control and R644C patient cell lines treated with the indicated increasing concentrations of lopinavir (LPV). Proteins were analyzed by SDS-PAGE and western blotting with α-prelamin A antibodies. (g) Quantification of samples in (f). Band intensity was quantified using Licor Image Studio Lite. Three independent protein lysates were blotted and analyzed. Values are means (*n* = 3), error bars are SEM.

For immunoblotting, lysates from ~1 × 10^4^ cells/lane were resolved by 10% SDS-PAGE and proteins were transferred onto a nitrocellulose membrane using the Bio-Rad Trans-Blot® TurboTM semi-dry transfer system. The membrane was blocked in 5% nonfat dry milk in PBS-Tween (PBST, which is 10% 10X homemade Phosphate Buffered Saline (8% NaCl, 0.2% KCl, 1.7% Na_2_HPO_4_, 0.24% KH_2_ PO_4_ , pH 7.4) with 0.1% Tween-20 and 10 mM NaN_3_) at room temperature for 1 hr, washed with PBST, incubated overnight at 4°C with primary antibodies, washed 3 times for 10 min with PBST, and incubated with the appropriate secondary antibodies for 30 min followed by washing with PBST (3 × 10 min each). Signals were detected on the Odyssey CLx imaging system using Image Studio Lite Software (LI-COR).

Lamin proteins were detected using mouse monoclonal anti-Lamin A/C antibodies (Santa Cruz, clone E1 (#sc -376248), 1:1000 dilution) and decorated with donkey anti-mouse secondary IRDye 800CW antibodies (LI-COR). Prelamin A was detected using rat monoclonal anti-prelamin A antibodies (clone 3C8, a generous gift of Drs. Loren Fong and Stephen Young, UCLA, 1:2000 dilution) and decorated with goat anti-rat secondary IRDye 680RD antibodies (LI-COR). ZMPSTE24 was detected using rabbit polyclonal anti-ZMPSTE24 antibodies (Invitrogen, #PA1–16965- lot number UD2763487, 1:1000 dilution). The HeLa wild-type (WT) and ZMPSTE24 knockout (KO) cell extracts used to validate ZMPSTE24 antibodies were generated as previously described [53]. β-Actin was detected using rabbit monoclonal anti-β-actin (Cell Signaling, clone 13E5, 1:1000 dilution). The latter two primary antibodies were decorated with goat anti-rabbit IRDye 800CW (LI-COR) secondary antibodies. All secondary antibodies were used at 1:10,000 dilution.

### Fixation of cells and indirect immunofluorescence (IF)

For fixation and staining procedures, cells were split and transferred from 6-well plates into three 24-well plates with a coverslip in each well, and propagated overnight. Fixation and staining were performed at room temperature. Cells on coverslips were fixed in 4% freshly prepared paraformaldehyde for 20 min, permeabilized in Triton permeabilizing solution (PBS pH 7.4, Gibco, with 0.1% Triton X-100) for 10 min, blocked in Triton permeabilizing solution with 3% BSA (Sigma, A2153) for 1 hr. Fixed and blocked cells then were sequentially incubated in primary and secondary antibody solutions in Triton permeabilizing solution for 1 hr each and stained with DAPI (5 μg/ml in PBS) for 10 min. Coverslips with the fixed and stained cells were dried and mounted on microscope slides using mounting solution (ProLong Antifade; Invitrogen, P36934). Slides were stored at 4°C until needed for imaging. The same primary and secondary antibodies were used as described for Westerns above, except that for immunostaining the mouse α-Lamin A/C antibodies were used at 1:500 dilution and α-mouse secondary antibodies used at 1:300 dilution.

### Image acquisition and data analysis

For counting nuclei, images were acquired by Leica/3i Spinning Disk Confocal Microscope using 40×/1.30 DIC oil immersion objective at a single focal plane. Random 40× microscopic fields were imaged to get at least 250 nuclei per coverslip/experiment. Images were processed using Zen Black edition software. The number of abnormal nuclei was determined by manual count and expressed as a percentage of total nuclei per experiment. Nuclear morphology was assessed as previously described [38,54]. In general, ‘normal’ nuclear morphology includes such features such as ovoid shape with few or no bulges and a smooth contour, with few folds or wrinkles, while ‘aberrant’ nuclear morphology is characterized by any of several features, including irregular shape, prominent blebs, folds, wrinkles, or nuclear ruptures, as illustrated in Figures 2 and 3. It should be noted that after FTI treatment, we observed the appearance donut-shaped nuclei in all cell lines, a phenomenon that has been previously described [55]. Donut-shaped nuclei were present in low and roughly equivalent percentage in all FTI-treated cells, independent of whether the cells were from healthy or affected individuals.

Approximately 350 nuclei were counted for wild-type (WT), HGPS, and MAD-B fibroblasts. For APS and R664C cells, ~250 were counted. Experiments were performed in triplicate and the standard deviation was calculated using Excel software STDEV.P function. Data were plotted as bar graphs. Student’s *t* tests of comparisons between two means were conducted using Excel. Representative images shown in Figures 2 and 3 were acquired by Carl Zeiss LSM confocal microscope using EC ‘Plan-Neofluar’ 40×/1.30 Oil DIC oil immersion objective at a single focal plane.

### Lonafarnib and lopinavir treatment

For lonafarnib treatment, cell lines at passage 14 (except PSADFN317, which was at passage 11) were seeded at approximately 7 × 10^4^ cells/well density into 6-well plates and allowed to attach and grow overnight. Three separate wells/experiments were designated for each treatment. On the next day the cell culture medium was changed with medium containing 0.1% DMSO (vehicle control), 2 μM lonafarnib was added (from a 100× stock in 100% DMSO) and the cells were incubated for an additional 48 hrs. After 48 hrs, the cells in each well were trypsinized and washed, as above, counted, and seeded at ~15,000 cells/well density into 24-well plates containing glass coverslips. Cells were allowed to attach ~8 hrs in medium with vehicle control or DMSO or 2 μM lonafarnib and subjected to IF. Lonafarnib was obtained from the PRF Cell and Tissue Bank.

For testing prelamin A accumulation after ZMPSTE24 inhibition, WT and R644C patient fibroblasts were seeded in 6-well plates as described above, then treated with medium containing 0.1% DMSO (vehicle control), or increasing concentrations of lopinavir (1, 2.5, 5, 10 or 20 μM) for 12 hr. Lysates were prepared as described above and analyzed by SDS-PAGE and Western blotting for lamin A/C and prelamin A. Lopinavir was obtained from NIH AIDS Reagent Program, Division of AIDS, HIAID, NIH.

## Results

### Mutations in patient fibroblasts analyzed in this study

A schematic of the final step of prelamin A processing is shown in Figure 1(a). In WT cells, the farnesylated C-terminal 15 amino acids of prelamin A are cleaved by ZMPSTE24 to produce non-farnesylated mature lamin A (Figure 1(a), left). In the progeroid laminopathies HGPS, MAD-B, and RD, processing fails to occur, and cells accumulate a disease-specific permanently farnesylated form of prelamin A (Figure 1(a), right). The first part of this study focuses on three MAD-B patient fibroblasts in which prelamin A processing is predicted to be defective, due to two different mutations in *ZMPSTE24*, P248L and -L425P (Table 1). Both missense mutations are found near, but not in, the ZMPSTE24 catalytic domain which contains a zinc ion, as shown in the structure (Figure 1(b)). MAD-B fibroblasts P248L–1 and P248L–2 are from siblings; they are heterozygous and carry a *ZMPSTE24*-P248L missense mutation and a *ZMPSTE24* null allele. The other MAD-B fibroblast, *ZMPSTE24*-L425P, is derived from a patient homozygous for this missense mutation.

Additionally, we examine four APS patient cells with mutations in *LMNA* (E138K, D325N, M540T, and C558R; Table 1) whose processing is expected to be normal, since their mutations lie at various positions in prelamin A, but none are near the processing site (Figure 1(c)). E138K, D325N, and C558R are heterozygous, while M540T is homozygous, and all appear to be dominant missense mutations that cause various levels of progeroid-like symptoms in the patients from which they are derived. Also included in our analysis as controls are fibroblasts from an HGPS patient, and an unaffected healthy individual (WT). All patient cells studied here and their sources are listed in Table 1. Clinical data for patients not previously reported is provided in the Supplemental Material.

### MAD-B patient cells accumulate prelamin A, while APS patient cells do not, but all exhibit varying levels of abnormal nuclear morphology

We predicted that MAD-B patient cells, whose mutations map to *ZMPSTE24*, would exhibit a prelamin A processing defect, while the APS patient cells would not, since the altered residues in *LMNA* in the latter are distant from the ZMPSTE24 cleavage site in prelamin A. To date, only mutational changes in prelamin A that lie at or quite close to the ZMPSTE24 cleavage site are known to alter processing [52,56].

To confirm the expected prelamin A processing profiles of MAD-B and APS patient cells, protein extracts were analyzed by SDS-PAGE and immunoblotting with α-lamin A/C (Figure 2(a), top panel) and α-prelamin A antibodies (Figure 2(a), second panel). In cells from a healthy individual (WT, lane 1), lamin A and C are the sole *LMNA* species evident. Little or no prelamin A is detectable, because conversion of prelamin A to mature lamin A is rapid. The four APS cell lines tested here show the same pattern as WT, namely only lamin A and lamin C are present (lanes 5–8). However, in the MAD-B cells (lanes 3 and 4) while lamin C is present, the upper band is actually prelamin A. It has a slightly slower gel mobility than lamin A (Figure 1(a), top panel) and stains with α-prelamin A antibodies (Figure 1(a), second panel). SDS-PAGE analysis for the MAD-B cell line P248L–1 is presented in Supplementary Material, Fig S1; the mobility shift and prelamin A staining is also evident in these cells. The HGPS fibroblast cell line, included as a control in this study (lane 2), shows the expected pattern of lamin A and lamin C staining, but also the presence of the progerin (∆50), which migrates between lamin A and C; there is no prelamin A staining, since most of the epitope recognized by the α-prelamin A antibody is missing in progerin.

We also evaluated these patient cell extracts for ZMPSTE24. Because antibodies that recognize ZMPSTE24 can vary greatly in efficacy depending on their source and even lot number, we confirmed the antibodies used here by immunoblotting extracts from WT HeLa cells and HeLa cells with a ZMPSTE24 knockout (KO) allele (Figure 1(b)). As previously seen [53], ZMPSTE24 runs as a doublet; the molecular differences between the two bands have not been determined and the ratio between them can vary in different cell lines. The presence of the doublet in WT cells and its absence in the ZMPSTE24 KO HeLa cells validates the α-ZMPSTE24 antibodies used in this study. Immunoblotting our panel of patient extracts with the α-ZMPSTE24 antibodies reveals that ZMPSTE24 is present in WT, HGPS, and APS samples, but notably absent in the MAD-B patient samples (Figure 2, third panel from top). The nearly complete absence of ZMPSTE24 in these patient cells at first might seem surprising, since all have at least one full-length copy of *ZMPSTE24*, albeit with a single substitution mutation. However, we previously found in a humanized yeast system that certain MAD-B alleles (including P248L and L425P) can affect not only ZMPSTE24 activity but also its protein stability [26,33]. That study suggested that these mutations cause misfolding, resulting in degradation of the mutant forms of ZMPSTE24 by the ubiquitin-proteasome system. Degradation likely explains why ZMPSTE24 is lacking, or at least strongly diminished, in the P248L and L425P patient cells here. A more extensive commentary on α-ZMPSTE24 antibodies, the ZMPSTE24 doublet, and protein instability caused by ZMPSTE24 disease alleles is included in the ‘Discussion’ section.

We examined nuclear morphology in our patient fibroblast panel (Figure 2(c)), since abnormal nuclear shape is known to be a hallmark of many *LMNA-* and *ZMPSTE24*-linked laminopathies. Most of the WT nuclei have a generally ovoid shape with relatively uniform lamin A/C staining and are devoid of irregularities. In contrast, as seen in representative fields from the patient fibroblasts examined here, all of them, including HGPS, MAD-B, and APS patients, had various striking abnormalities, including wrinkles, blebbing, folds, micronuclei and/or ruptures (Figure 2(c)). When quantitated, each disease cell line had highly increased percentages of nuclear shape abnormalities when compared to WT (Figure 3(a,c) solid bars).

Taken together, the analysis above of our panel of patient cells provides a firm foundation for further studies. While partial characterization of some nuclear phenotypes or SDS-PAGE analysis for patient cells with two of these mutations (*LMNA*-M540T and -C588R) has been previously published [51,57,58], here we present for the first time a systematic analysis of these, as well as the *LMNA* mutations E138K and D325N and the *ZMPSTE24* mutations P248L and L425P.

### The FTI lonafarnib improves nuclear morphology in MAD-B patient cells in which processing is defective, but not in processing-proficient APS cells

Treatment of HGPS fibroblasts with the farnesyltransferase inhibitor (FTI) lonafarnib is known to improve their nuclear morphology and lonafarnib is currently in use as a treatment for HGPS patients to great effect [45]. Generally, fewer studies have been conducted to evaluate the impact of FTI treatment on nuclear morphology in cells from other laminopathies. Importantly, one study concluded that MAD-B fibroblasts did not benefit from FTI treatment [48], which was surprising, since permanently farnesylated prelamin A is thought to be the disease culprit and FTI treatment prevents farnesylation. That report motivated studies to revisit this issue. Here we examined whether lonafarnib can be beneficial for improving MAD-B patient fibroblasts (Figure 3(a,b)). In addition, we examined lonafarnib’s effect on APS fibroblasts (Figure 3(c,d)). We did so because it has been suggested that preventing farnesylation of CAAX proteins that interact with or influence lamin A might potentially benefit a broader group of patients than just the processing defective laminopathy patients.

MAD-B patient cells P248L–1, P248L–2, and L425P, along with positive and negative controls (HGPS and WT, respectively) were treated with 2 µM lonafarnib or vehicle only (DMSO) for 48 hr. We counted the number of abnormal nuclei in each sample (~350 nuclei for each) and the experiment was conducted in triplicate. As expected, WT fibroblasts have a low percentage of abnormal nuclei (~20%), with and without lonafarnib (Figure 3(a)). In contrast, HGPS fibroblasts have a high percentage of abnormal nuclei (>70%) and exhibit a significant (~30%) decrease when treated with lonafarnib. All three MAD-B fibroblasts (P248L–1, P248L–2, and L425P) also have a higher percentage of abnormal nuclei than WT (ranging from 60% to 75%) and all exhibit a significant (10–22%) decrease in abnormal nuclei after lonafarnib treatment (Figure 3(a)). Representative immunofluorescence images are shown in Figure 3(b), to illustrate the trend for improved nuclei upon FTI treatment. Overall, this finding lends support to the concept that FTIs will likely benefit MAD-B patients defective for prelamin A processing. In the ‘Discussion’ section, we suggest possible reasons why our results differ from those of Akinci *et al*. [48], who concluded that FTIs did not improve nuclear morphology in P248L MAD-B patient cells.

A distinctly different picture emerges from analysis of the four APS patient fibroblasts (Figure 3(c,d)). While all show aberrant nuclear morphology (60–80%), after treatment with lonafarnib and quantification of ~250 nuclei in triplicate, none showed a significant improvement in nuclear morphology. One of the cell lines (M540T) even exhibited a significant increase in aberrant nuclear morphology with FTI treatment, for reasons we do not understand. Our finding that FTI treatment is not beneficial for APS fibroblasts suggests that FTIs are not likely to be useful as a treatment for APS patients.

### Two R644C patient fibroblasts from unrelated individuals exhibit no apparent processing defect and have normal nuclear morphology

The mutation R644C is a relatively common *LMNA* mutation and is notable for the extreme phenotypic diversity seen in *LMNA*-R644C patients and its variable penetrance within families [59]. The R644C mutation alters an arginine residue 3 amino acids upstream of the ZMPSTE24 cleavage site between residues Y646 and L647 in prelamin A (Figure 4(a)). It has been suggested that because the position of the R644C mutation is so close to the processing site, it may disrupt processing. However, data for prelamin A processing in R644C patient cells have not been previously reported. Studies examining the effect of the R644C mutation in the context of recombinant forms of prelamin A in HEK293 cells or yeast are conflicting [56,60]. A previously reported *LMNA* patient mutation, L647R, is adjacent to the cleavage site (indicated in Figure 4(a)) and causes a MAD-B-like disorder. In L647R patient fibroblasts, prelamin A processing is blocked and abnormal nuclear morphology is apparent [52]; these patient cells are used as a control here.

We examined the prelamin A processing profile in two unrelated *LMNA*-R644C patient fibroblasts, R644C-1 and R644C-2, including WT, HGPS, and L647R cells as controls. While prelamin accumulation is apparent in the L647R fibroblast control, no prelamin A is evident in the R644C cells, as also is the case for the WT and HGPS controls (Figure 4(b)), indicating that prelamin A processing is unaffected in R644C fibroblasts.

We also examined nuclear morphology in these patient cells. While altered nuclear morphology is apparent in HGPS and L647R fibroblasts, nuclear morphology is normal in R644C cells, as shown in representative examples (Figure 4(c)) and by nuclear counts (Figure 4(d)), where there is a small, but not significant, difference discernable between WT and R644C patient cells. Nor does lonafarnib treatment have a discernable effect on nuclear morphology (Figure 4(d)). Because HGPS fibroblasts accumulate an increasing amount of progerin during cell passage [21], we also asked if prelamin A might accumulate during passaging of R644C cells. However, no prelamin A was apparent in either early, middle, or late passages of R644C patient fibroblasts (Figure 4(e)).

We also considered the possibility that R644C cells might be ‘on the borderline’ for prelamin A processing and thus more sensitized to small fluctuations in ZMPSTE24 levels, as compared to WT cells. However, using the ZMPSTE24 inhibitor lopinavir [61,62] at increasing dosage and monitoring the level of prelamin A, no difference is apparent between WT and R644C cells in either the onset or rate of prelamin A appearance (Figure 4(f,g)). Thus, the R644C mutation does not appear to affect prelamin A processing, at least in the patient cells tested here.

## Discussion

In this study, we examined prelamin A processing and correctability of aberrant nuclear morphology by FTI treatment in cell lines from patients with *ZMPSTE24* and *LMNA* mutations. We found that MAD-B patient cells with mutations in *ZMPSTE24* accumulate prelamin A, as expected, and have abnormal nuclei which can be corrected with the FTI lonafarnib. We did not observe FTI correctability for cells from patients with non-HGPS *LMNA* mutations, referred to here as APS cells (see below). Lonafarnib was recently FDA-approved under the trade name Zokinvy and is supplied by Eiger Biopharmaceuticals, Inc [63,64]. It is in current use for treatment of HGPS based on clinical trial outcomes [45], and also for processing-deficient laminopathies. The latter indication is based in part on the pre-published preliminary results of this study. Although clinical trials have not been conducted specifically for MAD-B patients, our pre-published results and the fuller study presented here, along with mechanistic considerations, suggest that individuals with MAD-B, like those with HGPS, are likely to benefit from this drug. The preliminary results for the current study contributed to evidence supporting the FDA-approved indication for Zokinvy for patients with ‘processing-deficient progeroid laminopathies’ [63,64] and to the limitation stated in the label, that it is not advised for patients with processing-proficient laminopathies.

Our finding that the aberrant nuclear morphology of MAD-B cells is corrected by lonafarnib contradicts the conclusions of a report by Akinci *et al*. [48], but was foreshadowed by previous work in *Zmpste24-/-* mice and MEFs derived from them. Like MAD-B patient cells, *Zmpste24-/-* MEFs accumulate permanently farnesylated prelamin A and have abnormal nuclear morphology that improves with FTI treatment [39]. Untreated *Zmpste24*-/- mice are quite sick, but benefit from treatment with FTIs, with longevity and other symptoms improving significantly [47]. FTI treatment was also shown to improve nuclear morphology in an RD patient cell line [39] and in patient and mouse cells with *LMNA*-L647R and *LMNA-*L648R mutations, respectively, that express an uncleavable form of prelamin A [52,54]. Importantly, our findings here with MAD-B patient cells are in direct contrast to the results reported in a study by Akinci *et al*. [48] that concluded that there was no improvement in MAD-B patient cells with FTI treatment. The disparate results reported by our group and that of Akinci *et al*. are not due to patient cell-specific differences, since two of the three patient cell lines studied here (*ZMPSTE24*-P248L–1 and -P248L–2) are the very same as those examined by Akinci and coworkers (called MAD.B 4700.3 and 4700.4 in the Akinci *et al*. study [48] and in a previous clinical report by Ahmad *et al*. [34]). These patient cells were provided to the PRF Cell and Tissue Bank, where they are called PSADFN 317 and 318 and are the cells used herein (Table 1). Differences in the results reported here and in the study by Akinci *et al*. [48] could reflect the use of different FTIs, (lonafarnib in our study *versus* FTI-277 in Akinci *et al*.) or subtly different growth conditions. Taken together, the weight of evidence reported here and in other studies (with the exception of Akinci *et al.* [48]) suggests that lonafarnib will benefit MAD-B patients. Because RD infants are quite ill and succumb to disease perinatally, it is unlikely that the short time window available for treatment would be sufficient to see improvement in these patients.

In this study, we also examined patient cells with mutations throughout *LMNA*, but at a distance from the prelamin A processing site (E138K, D325N, M540T, and C588R). All were processing proficient, as also previously reported for cells from different patients with M540T and C588R mutations [51,58]. All showed abnormal nuclear morphology, some with quite interesting patterns, such as prevalent nuclear ruptures for M540T (Figure 2). However, as we expected, since none of these patient cells show accumulation of farnesylated prelamin A, none show correction of aberrant nuclear morphology by lonafarnib treatment. A previous study with *LMNA*-D300G patient cells, which are also processing-proficient, unexpectedly showed correction of aberrant nuclear morphology with FTI treatment [65]. The authors speculated that preventing farnesylation of a protein that acts with or downstream specifically of the D300G mutant version of lamin A could blunt the negative effect specifically of the D300G form of lamin A. Notably, for *LMNA*-D300N patient fibroblasts, FTIs did not improve cellular phenotypes [66]. Taken together, we expect based on the results here and elsewhere, that FTIs will benefit processing-defective laminopathies, but generally will not be useful for most of those laminopathies in which processing is proficient. We note that a limitation of the present study is that nuclear morphology was the sole phenotype analyzed. Future studies to test the efficacy of FTIs or other drugs might benefit from measuring correction of additional phenoytpes associated with permanently farnesylated forms of lamin A, such as DNA damage, loss of heterochromatin, or accelerated senescence.

We also examined here for the first time prelamin A processing in fibroblasts from two unrelated patients with a *LMNA*-R644C mutation, among the most common of disease-causing *LMNA* mutations. The *LMNA*-R644C allele is notable for both its extreme phenotypic diversity and its variable penetrance within single families [59]. At least 50 individuals with the R644C mutation have been reported in the literature and are listed in the Universal Mutation Database (http://www.umd.be/LMNA/). These individuals have been diagnosed with wide range of illnesses and symptoms, including APS, dilated cardiomyopathy (as is the case for the R644C-2 patient reported here), lipodystrophy, muscular dystrophy, and other disorders, while some individuals who carry the mutation appear to be unaffected. The fact that diagnoses differ even within a single family has suggested that additional genetic or physiological modifiers may impact the function of *LMNA*-R644C [59]. The individual who provided R644C-1 cells to the Progeria Research Foundation was apparently over 75 years old at the time, and the individual who provided R644C-2 cells showed no clear progeria symptoms.

The proximity of R644 to the ZMPSTE24 cleavage site 3 amino acids away, between residues Y646 and L647, has led to the suggestion that the R644C mutation may affect prelamin A processing. Until the present study, the status of prelamin A processing in R644C patient cells has not been heretofore documented by immunoblotting with prelamin A antibodies, in part due to the limited availability of fibroblast lines with this mutation, although a verbal description by one group reported that processing is normal in one R644C patient cell line [39]. In our own studies using recombinant systems, we have observed conflicting results on the impact of R644C on processing: On the one hand, when a GFP-tagged lamin A construct containing the C-terminal 51 residues of prelamin A with an R644C mutation was expressed in HEK293 cells, a partial processing defect was observed; however, a high level of expression in a small proportion of transiently transfected cells may have overwhelmed ZMPSTE24’s cleavage capacity in those experiments [60]. On the other hand, in a humanized yeast system where we systematically assessed prelamin A processing for *LMNA* mutations in the six residues surrounding the cleavage site, the *LMNA*-R644C mutant showed little or no processing defect [56]. However, patient fibroblasts provide a more physiological system for analysis. And because we clearly document that there is no defect in prelamin A processing for the two R644C patient fibroblast lines studied here, we suggest that the R644C mutation likely promotes disease because the single amino acid change from arginine to cysteine causes lamin A to gain or lose some of its normal function, as is also the case for many other laminopathy mutations, including C661S [67,68]. In the two patient cells we examined here no nuclear abnormalities were observed, however, aberrant nuclear morphology has been reported (although counts were not provided) for the single R644C patient cell heretofore available (from the Coriell Cell Repository) [39,69]. In any case, because prelamin A processing appears to be normal in the patient fibroblasts, it seems unlikely that *LMNA*-R644C patients would benefit from Zokinvy treatment, although it is conceivable that a processing defect might occur in some R644C patient cells other than fibroblasts.

This study also sheds new light on the status of ZMPSTE24 in MAD-B patient cells. MAD-B is caused by a partial loss-of-function allele in one copy of *ZMPSTE24* and a null allele in the other (as for the *ZMPSTE24*-P248L–1 and −2 cells studied here), or two partial loss of function alleles (as for the homozygous *ZMPSTE24*-L425P patient in this study). Here we showed that as expected, processing is defective and prelamin A accumulates in these patient cells, nuclear morphology is abnormal, and, as noted above, all were correctable by lonafarnib. Our studies uncovered another new facet of these cells: Even though they all have a single amino acid substitution mutation in *ZMPSTE24* (P248L or L425P), no or very little of the ZMPSTE24 protein is evident by Western immunoblotting. This might not be expected, since single point mutations often leave protein levels unaffected. However, we previously used a humanized yeast system to examine the activity and stability of mutant forms of ZMPSTE24 encoded by disease alleles. Interestingly, many but not all, of the *ZMPSTE24* disease alleles affected protein stability, including P248L or L425P. In that study we showed that the *ZMPSTE24*-P248L protein was degraded by the ubiquitin-proteasome system, presumably due to protein misfolding caused by the amino acid substitution. Notably, in the humanized yeast system *ZMPSTE24*-P248L could be stabilized, yielding improved prelamin A processing, by treating cells with the proteasome inhibitor bortezomib [26]. Bortezomib, also called Velcade, is FDA approved for treatment of multiple myeloma and mantle cell lymphoma [70]. It would be of interest to determine if bortezomib on its own and/or in combination with the FTI lonafarnib might be beneficial for patients with mutant alleles of *ZMPSTE24* like the P248L and L425P that appear to cause ZMPSTE24 degradation.

Regarding tools to study of MAD-B patient cells, it is worthwhile noting that very few commercially available anti-ZMPSTE24 antibodies actually recognize ZMPSTE24, when put to the test of immunoblot validation as shown in Figure 2(b), comparing WT and ZMPSTE24 KO HeLa cells (or MEFs from WT or *Zmpste24*-/- mice) [16]. Our laboratory has tested numerous antibodies obtained from several companies. Of those we tested, only Invitrogen polyclonal rabbit α-ZMPSTE24, (#PA1–16965) showed a signal in WT cells that is absent in ZMPSTE24 KO cells (Figure 2). And even at that, not all lot numbers for this same antibody worked equally well, or even at all. For Figure 2 we used lot # UD2763487, which shows a strong signal for ZMPSTE24, but other lot numbers (*e.g*. lot #’s WH3347616, XA347066) from the same company do not show a signal (data not shown). Surveying the literature, it appears that an antibody against ZMPSTE24 from Daiichi Chemical worked well as validated by others [16,28], however these antibodies are no longer commercially available. Because of the variability in commercially available α-ZMPSTE24 antibodies, any study that uses these should also provide internal validation. We further note that in all cases where a validated ZMPSTE24 signal can be seen, ZMPSTE24 migrates by SDS-PAGE as a doublet, sometimes with the bottom band running as a smear as seen here and elsewhere [16,26,28,53]. These doublet bands do not reflect differences of the *N*- and C-termini of ZMPSTE24 as judged by epitope tagging experiments (data not shown) and thus may represent as yet undetermined differences in post-translational modifications of ZMPSTE24. Like many other multispanning membrane proteins ZMPSTE24 runs anomalously by SDS-PAGE. ZMPSTE24’s predicted molecular weight is ~55 kD, but the doublet bands migrate anomalously by SDS-PAGE at ~40–50 kD.

Finally, we wish to note that the terms ‘atypical progeroid syndrome’ (APS) used here, and ‘atypical HGPS,’ or ‘non-classic HGPS’, have been variously and often interchangeably used in the literature to describe laminopathies with clinical features that at least partially overlap with those of HGPS patients. Examples of overlapping symptoms between APS and HGPS include failure to thrive, short stature, similar facial features, lipodystrophy, acral osteolysis, skin atrophy, and/or hair loss alopecia [51,71]. Three of the patient mutations studied here (*LMNA*-E138K, -C588R, and -R644C) have been previously categorized as causing APS [51,69,71], and we use this terminology here. While classic HGPS refers to the *LMNA* exon mutation G608G, non-classic HGPS generally refers to nearby intron mutations, and both interrupt normal spicing, resulting in progerin (∆50) production. However, we caution that use of all of these terms (APS, atypical HGPS, and non-classic HGPS) may be confusing especially for laminopathy patients and their health care providers, because the word ‘progeria’ might suggest that FTI treatment will be beneficial for these patients. Thus, we suggest that clarification of terms used to refer to laminopathies and/or specific *LMNA* and *ZMPSTE24* mutations could be helpful for patients, their health-care providers, and researchers in the laminopathy field. As previously pointed out by Doubaj *et al*. [71], the molecular characterization of patient cells is important to optimally predict the efficacy of potential treatments. In this regard, the more mechanistic indication specified for the FTI Zokinvy, namely for treatment of ‘HGPS and processing-deficient progeroid laminopathies’, relies on appropriate mechanistic studies revealed by cellular and molecular testing, as shown in this study, to assess prelamin A processing.

## Supplementary Material

Supplemental Material

## Data sharing

The authors confirm that the data supporting the findings of this study are available within the article [and/or] its supplementary materials.
