# Supplementary material for: The farnesyl transferase inhibitor (FTI) lonafarnib improves nuclear morphology in ZMPSTE24-deficient fibroblasts from patients with the progeroid disorder MAD-B
Source: Nucleus. 2023 Dec 5;14(1):2288476. doi: 10.1080/19491034.2023.2288476 (PMC10730222; doi:10.1080/19491034.2023.2288476)
Supplement: Supplemental Material [file KNCL_A_2288476_SM4250.docx]

**SUPPLEMENTAL MATERIAL**

**Clinical Features of Patients**

A brief description of the clinical features of patients not previously published and listed in Table 1 as “This Study” is provided below. Clinical information for all of the patients except R644C-2 is from The Progeria Research Foundation’s Medical and Research Database and International Registry (www.progeriaresearch.org). PRF Cell and Tissue Bank and database are approved by the Hasbro Children's Hospital IRB and are consented programs. Cells and Information for R644C-2 are from Daniel P. Judge. R644C-2 cells were obtained after signed informed consent, and the research was approved by the Johns Hopkins University Institutional Review Board.

PSADFN485 (*LMNA-*E138K)

Patient information was submitted at age 3 years. The patient showed failure to thrive, with poor weight gain starting at ~9-12 months of age. Hair loss was apparent since infancy. The patient has pale skin with visible vasculature on the head. Growth of teeth was delayed until 13 months of age, and when they came in, teeth were overcrowded, crooked, and rotated. No joint problems were noted but some stiffness was detected. The patient passed away at 10 years of age, and the cause of death is unknown to PRF.

PSADFN363 (*LMNA*-D325N)

Patient information was first submitted at age 3 months. The patient was born at 36 weeks of gestation, with a weight of 1.9 kg. Intestinal malrotation was operated on at 2 days of age. The patient had no subcutaneous fat, micrognathia, clenched fingers, divergent strabismus, large neurocranium, failure to thrive, no eyelashes, prominent scalp veins, small ears, glaucoma, increased pigmentation, full cheeks, prominent eyes, prominent forehead. A physician’s clinical summary at age 5 months indicates persistent nutritional problems with great difficulties to tolerate oral feeding and multiple infectious complications. At 6 months of age the patient had septicemia during the summer and the intestines appeared to have diffuse necrotizing enterocolitis or inflammation and bloody stools were frequent. The patient passed away at age 9 months after rapidly worsening state of health.

PSADFN542 (*LMNA*-R644C); also called R644C-1 in this study

A clinical summary was submitted when the patient was 75 years of age. The patient was referred for un-diagnosed neurological issue. the patient has a history of significant tremor and obsessive-compulsive disease, remote alcohol use, chronic double vision, frequent premature atrial beats. Skin biopsy suggested small fiber neuropathy. Autonomic testing suggested autonomic neuropathy. White matter abnormality was noted on MRI of brain.

*LMNA*-R644C-2:

The patient is a woman who presented at age 40 years with hypertension, muscle weakness, and nonischemic dilated cardiomyopathy. Serum creatine kinase level was normal on presentation and 2 years prior. She was ambulatory with mild symmetric weakness in her arms, normal strength in her legs. Her echocardiogram showed LV dilation (6.2 cm) with moderate LVH (1.6 cm), and reduced ejection fraction (40%). There was mild mitral and tricuspid regurgitation without valve calcification or stenosis. Her ECG showed sinus rhythm without conduction delay.  Her LVEF improved with use of an angiotensin receptor blocker and beta-blocker. She had mild cardiomyopathy, and perhaps mild muscular dystrophy.

PSADFN373 (*ZMPSTE24*-L425P)

Patient information was submitted at 5 years of age. Symptoms seen in hair, skin, tooth delay, and knee joint problems. Problems noted include failure to thrive, skull vault deficiency, inability to bend knees, alopecia, sclerodermatous changes on skin. Mother and father are first maternal cousins. The child's younger brother has the same condition.

 
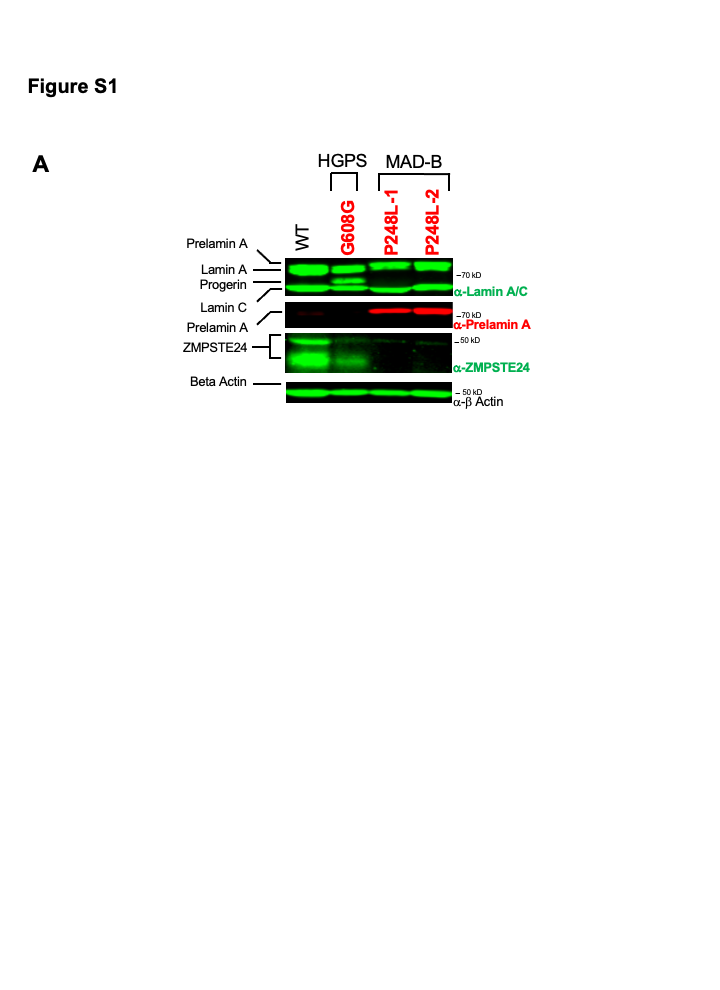


**Figure S1. Analysis of prelamin A processing status in patient cells used in P248L-1 and P248L-2.** Immunoblots of protein extracts from WT, HGPS, and MAD-B P248L-1 and P248L-2 patient fibroblasts.  Extracts from fibroblasts, prepared at passage 12 were analyzed by 10% SDS-PAGE transferred to nitrocellulose, and probed with the indicated antibodies. The panels from top to bottom show lamin A/C, prelamin A, ZMPSTE24, and β-actin staining, as indicated.
